# Supplementary material for: Consequences of the Lack of TNFR1 in Ouabain Response in the Hippocampus of C57BL/6J Mice
Source: Biomedicines. 2022 Nov 15;10(11):2937. doi: 10.3390/biomedicines10112937 (PMC9688030; doi:10.3390/biomedicines10112937)
Supplement: Supplementary file 1 [file biomedicines-10-02937-s001.zip › biomedicines-1935263-supplementary.pdf]

Supplementary

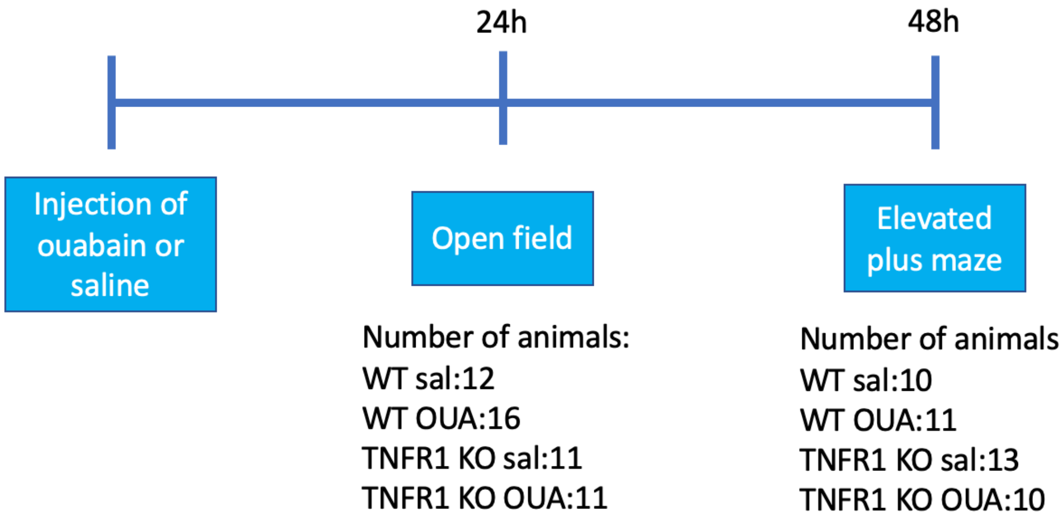

Figure S1. Scheme of the behavioral tests timeline. The open field test was performed 24 hours after the injection and the elevated plus maze after 24 hours after the open field test.

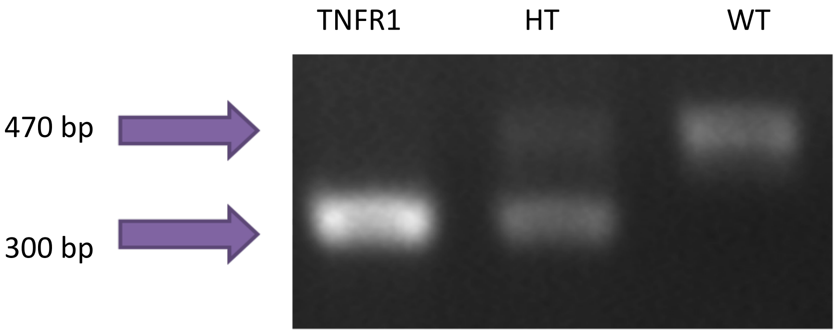

Figure S2. Genotyping of mice. The band of 300 base pairs (bp) corresponds to TNFR1 KO mice (TNFR1), 470 bp corresponds to WT mice and both bands represent heterozygous animals.

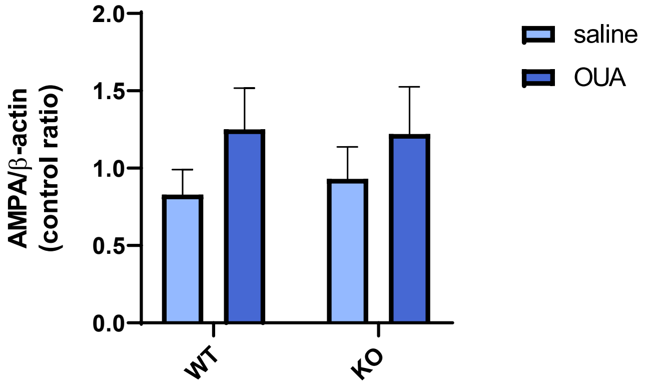

Figure S3. Ouabain treatment and TNFR1 does not impact in total AMPA expression. Results were expressed in control ratio from four individual experiments.

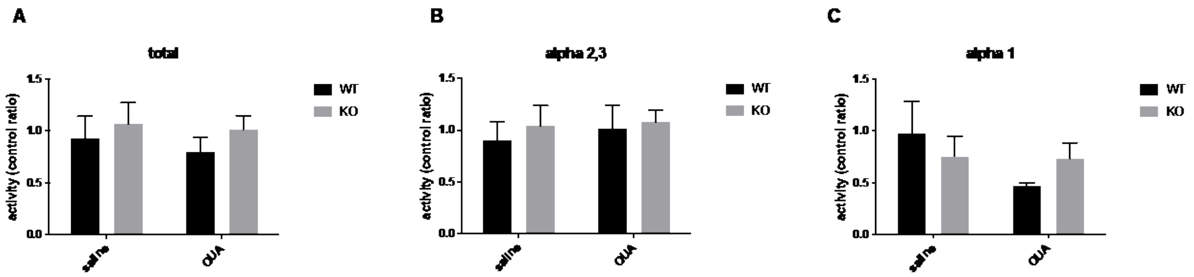

Figure S4. Ouabain treatment and TNFR1 does not impact in NKA activity concerning total activity (A), alpha 2 and 3 activity (B), and alpha 1 activity (C) in the hippocampus extracts. Results were expressed in relative activity by the control ratio from four individual experiments.
